# Varactor-tuned wideband band-pass filter for 5G NR frequency bands n77, n79 and 5G Wi-Fi

**DOI:** 10.1038/s41598-022-20593-x

**Published:** 2022-09-29

**Authors:** Alireza Golestanifar, Gholamreza Karimi, Ali Lalbakhsh

**Affiliations:** 1 Electrical Engineering Department, Faculty of Electrical and Computer Engineering , Razi University, Kermanshah, Iran 6714967346; 2 School of Engineering, Macquarie University, Sydney, NSW Australia; 3 School of Electrical and Data Engineering, University of Technology Sydney (UTS), Sydney, NSW 2007 Australia

**Keywords:** Electrical and electronic engineering, Engineering, Materials science

## Abstract

A wide-band band-pass filter (BPF) using coupled lines, rectangular stubs and Stepped-Impedance Resonators (SIRs) is presented in this paper. The proposed BPF operates over a large pass-band from 3.15 to 6.05 GHz covering 5G New Radio (NR) frequency Bands n77, n79 and 5G Wi-Fi, which includes the G band of US (3.3 to 4.2 GHz), 5G band of Japan (4.4 to 5 GHz) and 5G Wi-Fi (5.15 to 5.85 GHz). The presented filter has a maximum pass-band Insertion-Loss (IL) of 2 dB, a sharp roll-off rate and suppresses all the unwanted harmonics from 4.2 GHz up to 12 GHz with a 15 dB attenuation level. The performance of each section can be analyzed based on lumped-element circuit models. The electrical size of the BPF is 0.258 λ_g_ × 0.255 λ_g_, where λ_g_ is the guided wavelength at the central frequency. The design accuracy is verified through implementing and testing the final BPF. The pass-band band-width can be controlled by adding the varactor diodes. A good relationship between the band-width and the varactor diodes are extracted by the curve fitting technique.

## Introduction

The fifth generation of cellular networks (5G) new radio (NR) is a new Radio Access Technology (RAT) developed by 3rd Generation Partnership Project (3GPP) for the 5G mobile network. It was designed to be the global standard for the air interface of 5G networks. Bands n77, n79 and 5G Wi-Fi, which include the 5G band of US (3.3 to 4.2 GHz), 5G band of Japan (4.4 to 5 GHz) and 5G Wi-Fi (5.15 to 5.85 GHz). 5G technology needs high-tech methods to transfer waves with high data density^1–4^. To transmit or receive signals, for example at 1.5 GHz and 2 GHz, two major approaches can be used. In the first approach, multiple narrow-band communication devices are used to operate in each band. In the second method, wide-band communication devices are used along with microstrip filters to filter the frequency range of interest and attenuate other frequencies. A diplexer is a good choice to filter and choose bands but the delivered power will be decreased. For example, a two-channel diplexer will deliver only half of the total power injected to the diplexer, at the best situation. Alternatively, varactor-tuned microstrip filters can be used, especially for low power signals, for example in low noise amplifiers. This paper proposes a Varactor-tuned Wide-band Band-Pass Filter which can be used in 802.11.n/ac/ac/ax/be or 5G Wi-Fi. Some of the applications of these bands are transferring data from a base to a far Remote Radio Unit (RRU) and then transferring these data via n77, n79 to 5G mobile phones. Channel n77 and n79 can both have signal band-widths of 10 MHz to 100 MHz and signal is transferred via Time-division duplexing (TDD). Varactor tuned filters have many applications like cognitive radio and reconfigurable antennas^5–7^. These filters can be applied to suppress unwanted channels and frequency bands. Cognitive radio can detect which signal and channel are used and which one is not and can move on to a vacant channel or band-width. 5G communication systems need different frequency frames and band-widths. This is making microwave tunable filters an integral component in such modern systems. In^8^, a tunable dual-band BPF using a polygonal resonator was presented, where one of its transmission poles is independently tunable using a varactor diode. The substrate integrated waveguide (SIW) method is applied to reach a tunable microstrip BPF^9^. Resonance cavities in vertical and horizontal topology are applied. A coupling matrix was utilized to analyze the resonators. In^10^, SIRs are utilized to obtain a dual-band BPF. The SIR’s gap alteration improved both in and out-of-band responses of the filter. In other words, Transmission zeros get closer and vice versa. In^11^, three different, second-order BPF topologies with coupled microstrip structures were presented with no, one and three transmissions zeros. The structures were accompanied by stubs to suppress parasitic harmonics. In^12^, a tunable microstrip BPF with a fixed centre frequency was presented, where Positive Intrinsic Negative (PIN) diodes were utilized as switches (ON or OFF) to reach three different states with different band-widths. A ring resonator and folded short stubs were used to achieve a tuning range of 0.9 GHz to 2.2 GHz for a central frequency of 1.3 GHz. Microstrip band-stop resonance filters were utilized as suppressing cells to obtain wide-band BPF^13^. Adding mores suppressing cells will result in more IL in stop-band. In^14^, two wide-band BPFs are designed and combined via a coupling microstrip structure. In^15^, a microstrip BPF using stepped-impedance stubs were used to suppress parasitic harmonics. The filter has a Fractional-Band-Width (FBW) of 62% at the centre frequency of 5 GHz. In^16^, inter-digital microstrip resonators and inverters as high-pass and low-pass structures were combined to obtain a band-pass filter. The equivalent LC circuit and quasi-lumped-elements were used in^17^ to understand the transmission zeros behavior. Also, open and short subs were utilized to improve the upper stop-band up to the third harmonic of a microstrip BPF. A tunable dual-band microstrip BPF using stub loaded ring resonators was presented in^18^, where the key factor of the filter was independent tunable bands. In^19–21^, efficient coupling structures were applied to reach a wide pass-band, where even and odd analyses were utilized. The structure presents multiple transmission poles and zeros, which resulted in a sharp skirt factor and wide stop-band. Cross-shaped coupled microstrip resonator was applied in^22^ to reach two adjacent pass-bands as a dual-band BPF. Recently artificial intelligence-based approaches have been extensively used in microwave filters. Various types of nature-based algorithms, such as artificial intelligence algorithm^23–27^, particle swarm optimization^28–30^, Grey wolf optimization^31–33^, ant colony^34^ can be incorporated in the design procedures.

In this paper, a tunable wide-band microstrip BPF is presented. This filter only uses three varactor diodes installed at the end of the stubs. Two control parameters for the diodes to achieve the desired band-width with acceptable IL. Folded symmetric coupling structures were applied to create Radio Frequency (RF) chock and wide pass-band as the Fundamental Resonator (FR). To reach primary desired band-width specifications, such as central frequency and FBW of the filter, an equivalent LC circuit is used to extract transmission zeros and poles of the FR.

## Design process

### FR

**Figure 1:**
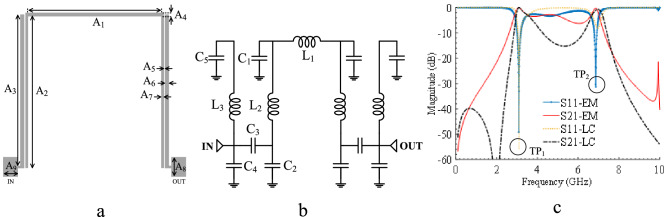
FR: (**a**) Layout, A_1_ = 10.7, A_2_ = 11.85, A_3_ = 12, A_4_ = 0.3, A_5_ = 0.1, A_6_ = 0.3, A_7_ = 0.2, A_8_ = 1.55 and A_9_ = 1.17 (unit: mm), (**b**) LC model, (**c**) EM and LC simulations.

One of the mainstream BPF design techniques is the use of coupled lines as the primary structure of the filter to provide the initial pass-band, which can be tailored later by various techniques^2,35–38^. In this work, two open-circuited stubs and a bended line are coupled, forming FR shown in Fig. 1a. The LC model of the FR is presented in Fig. 1b, justifying FR's filtering mechanism. In this model, L_1_, L_2_, C_1_ and C_2_ describe the inductances and capacitances of the bended line, respectively. C_3_ is the coupling capacitance between the open-circuited stubs and the bended line. Also, each open-circuited stub has a circuit model including L_3_, C_4_ and C_5_ as inductance and capacitances, respectively. The initial values of inductors and capacitors were calculated using the formulas explained in^2^ and then optimized by Advanced Design System (ADS) software as follows: L_1_ = 1.521 nH, L_2_ = 1.177 nH, L_3_ = 2.256 nH, C_1_ = 2 pF, C_2_ = 0.295 pF, C_3_ = 0.492 pF, C_4_ = 0.9 pF and C_5_ = 2.8 pF. The Electro Magnetics (EM) and LC simulations are illustrated in Fig. 1c. It should be mentioned that all components designed in this work were implemented using microstrip technology. It is shown that LC and EM model are in good agreement. Microstrip lines have different behavior at different frequencies and even more complex LC models cannot match exactly.

**Figure 2:**
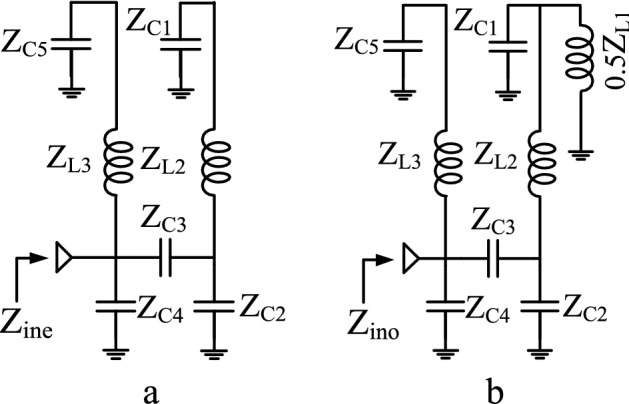
LC model of FR: (**a**) Even mode, (**b**) Odd mode.

**Figure 3:**
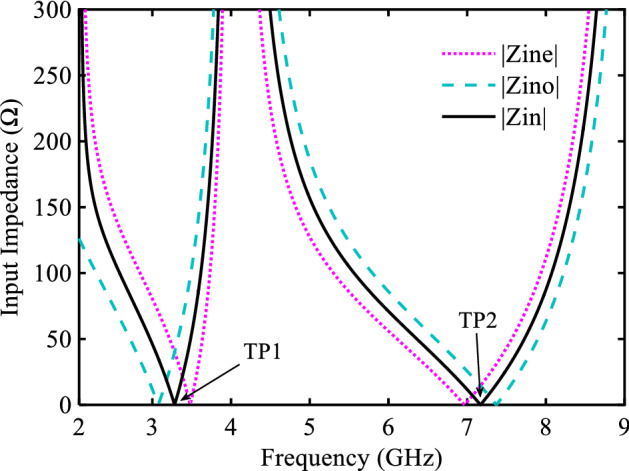
Input impedance of LC model of FR.

As observed, the FR produces two transmission poles (TP_1_ and TP_2_) at lower (3.08 GHz) and upper (6.86 GHz) edges of the pass-band that control the pass-band range, demonstrating a wide pass-band. The modal analysis is applied to calculate TP_1_ and TP_2_, parametrically. Even and odd modes of the LC model are demonstrated in Fig. 2a and b. The modal input impedances Z_ine_ and Z_ino_ (corresponding to (1) and (2), respectively) are obtained from Fig. 2. The input impedances of FR, including Z_ine_, Z_ino,_ and Z_in_ are plotted by MATLAB in Fig. 3. It can be seen that at the resonant frequencies, the input impedance of the FR is zero, and hence TP_1_ and TP_2_ are calculated by equating (3) and (4) to zero.

1 $$Z_{ine} = \frac{{(C_{1} + C_{2} + C_{3} + S^{2} C_{1} L_{2} (C_{2} + C_{3} ))(S(C_{4} + C_{5} ) + S^{3} C_{5} C_{4} L_{3} ) + (1 + S^{2} C_{5} L_{3} )(SC_{3} (C_{1} + C_{2} ) + S^{3} C_{1} C_{2} C_{3} L_{2} )}}{{(SC_{3} (C_{1} + C_{2} ) + S^{3} C_{1} C_{2} C_{3} L_{2} )(S(C_{4} + C_{5} ) + S^{3} C_{5} C_{4} L_{3} )}}.$$

2 $$Z_{ino} = \frac{\begin{gathered} \begin{array}{*{20}c} {} & {} & {} & {} \\ \end{array} (2 + S^{2} (C_{3} L_{1} + 2C_{3} L_{2} + C_{1} L_{1} + C_{2} L_{1} + C_{2} L_{2} ) \hfill \\ \begin{array}{*{20}c} {\begin{array}{*{20}c} {} \\ \end{array} } & {} \\ \end{array} + \;S^{4} (C_{1} C_{3} L_{1} L_{2} + C_{1} C_{2} L_{1} L_{2} ))(S(C_{4} + C_{5} ) + S^{3} C_{5} C_{4} L_{3} ) \hfill \\ + \;(2SC_{3} + S^{3} C_{3} (C_{1} L_{1} + C_{2} L_{1} + C_{2} L_{2} ) + S^{5} C_{1} C_{2} C_{3} L_{1} L_{2} )(1 + S^{2} C_{5} L_{3} ) \hfill \\ \end{gathered} }{{(2SC_{3} + S^{3} C_{3} (C_{1} L_{1} + C_{2} L_{1} + C_{2} L_{2} ) + S^{5} C_{1} C_{2} C_{3} L_{1} L_{2} )(S(C_{4} + C_{5} ) + S^{3} C_{5} C_{4} L_{3} )}}.$$

3 $$TP_{1} = \frac{1}{2\pi }\sqrt {\frac{{ - \sqrt {(C_{1}^{2} L_{1}^{2} + 2C_{1} C_{2} L_{1}^{2} - 6C_{1} C_{2} L_{1} L_{2} + 2C_{1} C_{3} L_{1}^{2} - 4C_{1} C_{3} L_{1} L_{2} + C_{2}^{2} L_{1}^{2} + \;2C_{2}^{2} L_{1} L_{2} + C_{2}^{2} L_{2}^{2} + 2C_{2} C_{3} L_{1}^{2} + 6C_{2} C_{3} L_{1} L_{2} + 4C_{2} C_{3} L_{2}^{2} + C_{3}^{2} L_{1}^{2} + \;4C_{3}^{2} L_{1} L_{2} + 4C_{3}^{2} L_{2}^{2} )} + \;(C_{1} L_{1} + C_{2} L_{1} + C_{2} L_{2} + C_{3} L_{1} + 2C_{3} L_{2} )}}{{2C_{1} L_{1} L_{2} (C_{2} + C_{3} )}}}$$

4 $$TP_{2} = \frac{{\sqrt {\frac{{\sqrt {(C_{1}^{2} L_{1}^{2} + 2C_{1} C_{2} L_{1}^{2} - 6C_{1} C_{2} L_{1} L_{2} + 2C_{1} C_{3} L_{1}^{2} - 4C_{1} C_{3} L_{1} L_{2} + C_{2}^{2} L_{1}^{2} + 2C_{2}^{2} L_{1} L_{2} + C_{2}^{2} L_{2}^{2} + 2C_{2} C_{3} L_{1}^{2} + 6C_{2} C_{3} L_{1} L_{2} + 4C_{2} C_{3} L_{2}^{2} + C_{3}^{2} L_{1}^{2} + 4C_{3}^{2} L_{1} L_{2} + 4C_{3}^{2} L_{2}^{2} )} + \;(C_{1} L_{1} + C_{2} L_{1} + C_{2} L_{2} + C_{3} L_{1} + 2C_{3} L_{2} )}}{{2C_{1} L_{1} L_{2} (C_{2} + C_{3} )}}} \cdot a + b}}{{4\pi \sqrt {C_{1} } \sqrt {L_{2} } \sqrt {C_{2} + C_{3} } }},$$

where

$$a = \sqrt {C_{1} } \sqrt {L_{2} } \sqrt {C_{2} + C_{3} } ,$$

$$b = \sqrt {C_{1} + C_{2} + C_{3} } .$$

**Figure 4:**
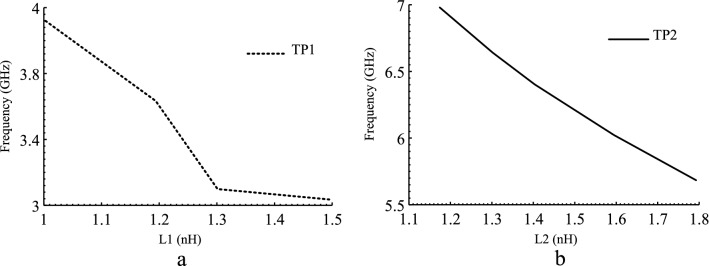
FR: (**a**) Variations of TP1 versus L1, (**b**) Variations of TP2 versus L2.

The pass-band range can be varied by relocating TP_1_ and TP_2_. From Fig. 4, the locations of TP_1_ and TP_2_ can be easily shifted by changing L_1_ and L_2_ elements, respectively. Since the BPF is to operate in 5G band, TP_1_ and TP_2_ are fixed at 3.08 GHz and 6.86 GHz. While FR demonstrates good band-width flexibility, the in and out of band performance does not meet the harsh 5G requirements.

### Proposed BPF

**Figure 5:**
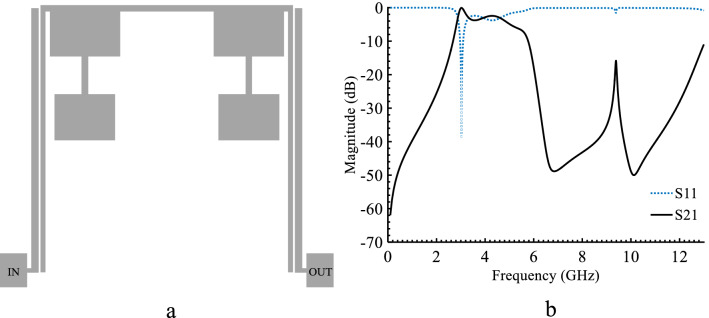
FR combining with SIRs: (**a**) Layout, (**b**) EM simulation.

**Figure 6:**
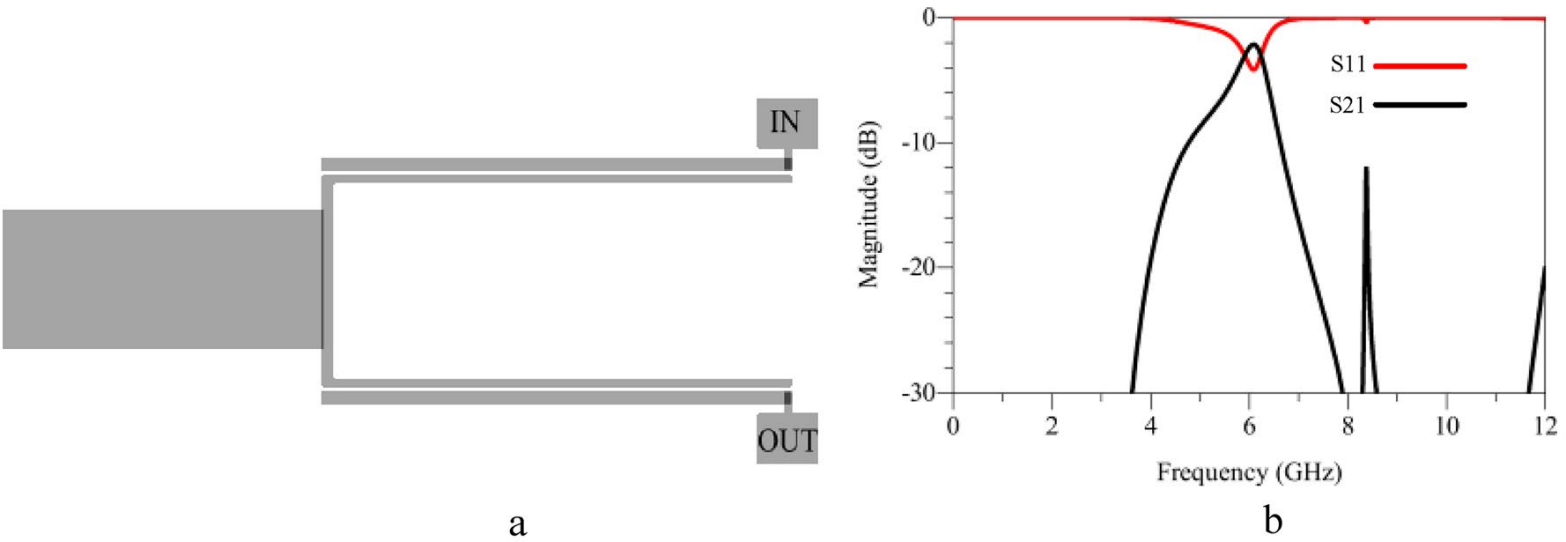
FR combining with rectangular stub: (**a**) Layout, (**b**) EM simulation.

**Figure 7:**
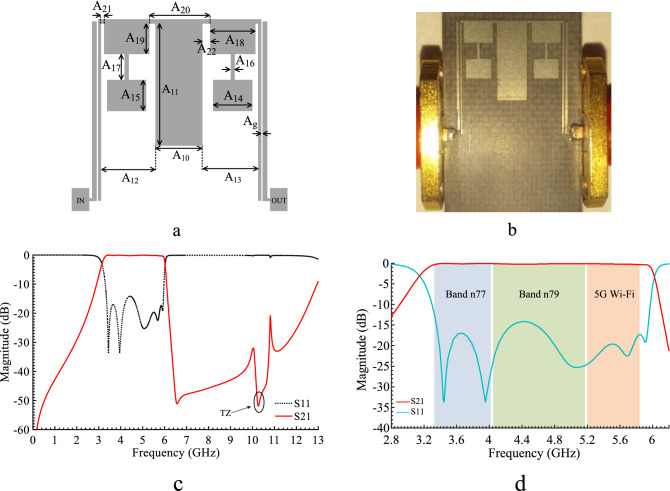
Proposed BPF: (**a**) Layout (**b**) prototype, (**c**) EM simulation result (**d**) Pass-band range EM simulation.

In order to significantly improve the quality of the FR response, more resonators need to be added to the circuit. This can be done by introducing a pair of SIR, as shown in Fig. 5a. The EM simulation of the structure is depicted in Fig. 5b showing a pass-band of 2.5–6 GHz, with an undesired IL (more than 5 dB) around 6 GHz. To improve the pass-band and reduce its ripples a rectangular stub is introduced. The layout and the EM simulation of the rectangular stub are depicted in Fig. 6a and b respectively. The EM simulation shows a good pass-band around 6 GHz. The final BPF is formed by connecting the FR, rectangular stub and SIRs to attain high performance and to suppress unwanted harmonics. The layout, fabricated prototype and the EM simulation results are shown in Fig. 7a–d. Figure 7a and b, respectively. The physical dimensions of the final structure are: A_10_ = 3.2, A_11_ = 8.15, A_12_ = 3.7, A_13_ = 3.7, A_14_ = 2.7, A_15_ = 2.1, A_16_ = 0.3, A_17_ = 1.7, A_18_ = 3.1, A_19_ = 2.05, A_20_ = 4.1, A_21_ = 0.2, A_22_ = 0.5, A_g_ = 0.1 (unit: mm). Figure 7c and d are illustrating wide and Pass-band range EM simulation results. These simulation results show that the proposed BPF has a wide stop-band from 6.5 GHz to 13 GHz with the IL of 20 dB and the pass-band is explained with more details. It is shown, a wide pass-band from 3.15 to 6.02 GHz is achieved.

**Figure 8:**
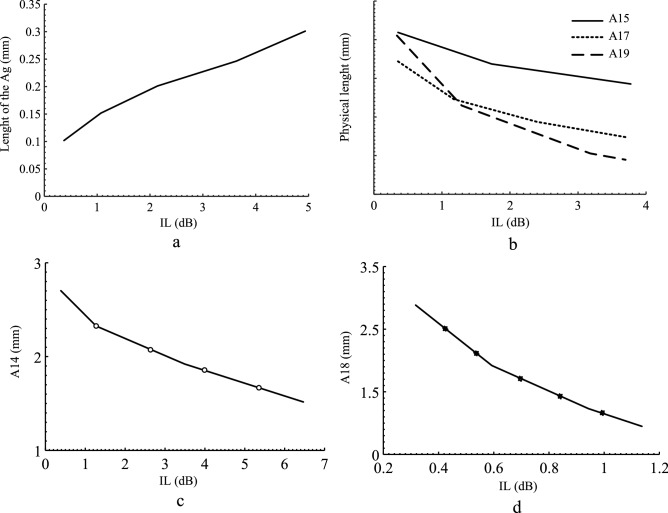
Proposed BPF: (**a**) Variations of IL versus A_g_, (**b**) Variations of IL versus A_15_, A_17_ and A_19_, (**c**) Variations of IL versus A_14_, (**d**) Variations of IL versus A_18_.

The primary structure (FR) is analyzed via even/odd mode analysis. This modal analysis shows the tunability of the FR and its relationship with parameters of the circuit. Then the two resonators are added to the FR to improve its pass-band IL. These two structures are then used for matching, tuning purposes and connecting the varactor diodes. The variations of the IL versus physical lengths of the proposed BPF are demonstrated in Fig. 8. From Fig. 8a, altering the gap (Ag) has a direct impact on the value of the IL, which means increasing Ag would increase IL up to 5 dB. Also, decreasing A15, A17 and A19 lengths can enhance the IL value in the pass-band, as seen in Fig. 8b. Reducing A14 and A18 would increase IL as depicted in Fig. 8c and d.

**Figure 9:**
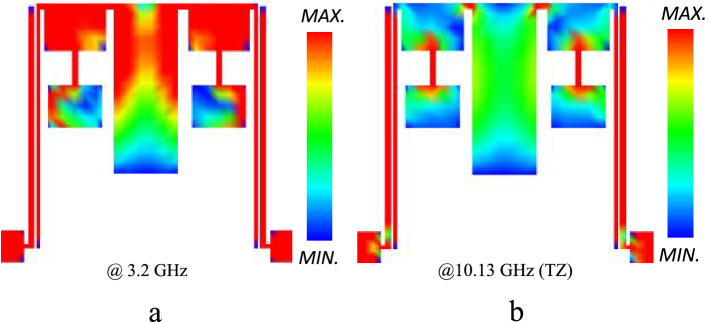
Current density distribution of proposed BPF: (**a**) 3.2 GHz, (**b**) 10.13 GHz.

The current density distribution is provided to get a better insight into the frequency behavior of the proposed filter. The current distributions were analyzed following the instructions in^39,40^ for two critical frequencies of 3.2 GHz (cut-off frequency) and 10.13 GHz (transmission zero). As shown in Fig. 9a, at 3.2 GHz the two introduce resonators contribute to the current flow on the top transmission line. On the contrary, both stepped-impedance and the rectangular resonators prevent the current at the transmission zero at10. 13 GHz, transmission zero, according to Fig. 9b. It can be observed that the strongest current flow occurs at modal peaks. Also, there is a poor density at TZ due to suppression of TZ to − 50 dB.

### Varactor diodes

**Figure 10:**
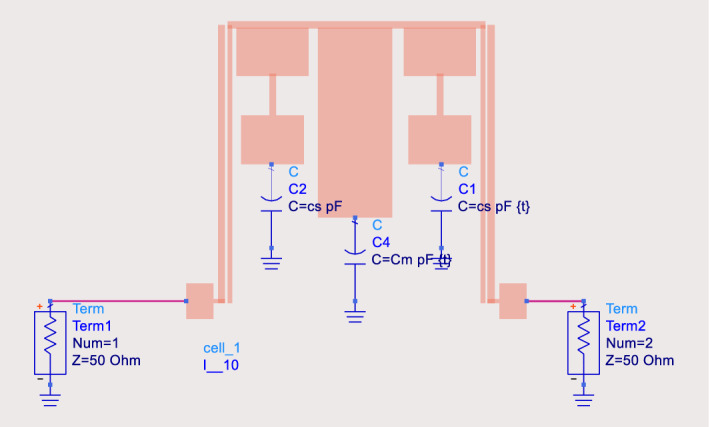
The proposed filter structure with PIN diodes.

To change the band-width of the filter designed in the previous section, three varactor diodes are used at the end of the stepped-impedance and the middle stub. Figure 10 shows the schematic of the proposed structures by adding the ideal diodes. Here for easy design process only capacitors are presented as the ideal diodes model.

**Figure 11:**
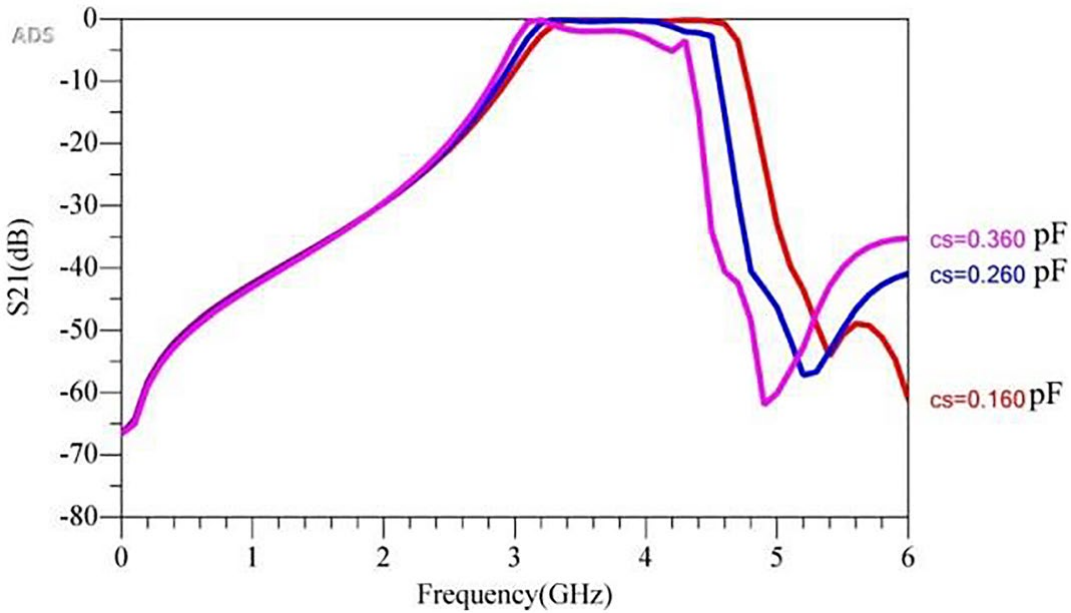
Frequency simulation response of the filter for various values of Cs.

Two side capacitors (Cs) can lower the upper cut-off frequency but the pass-band distort. Figure 11 illustrates the frequency simulation response of the filter for various values of Cs and a constant value of Cm = 0.5 pF.

**Figure 12:**
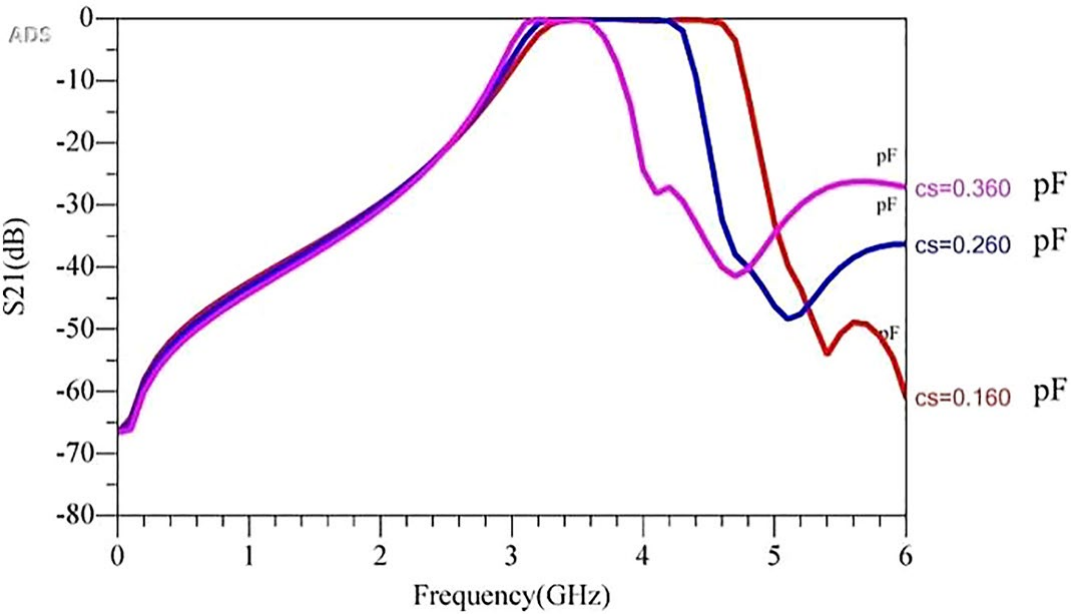
Frequency response simulation of the proposed filter for multiple optimized values of Cs and Cm.

It is seen that varying the values of Cs changes the upper pass-band of the filter; however, it results in a large IL. Also, there is a trade-off between IL and band-width as Cs values changes. It is seen that the IL is large for the case of Cm = 0.5 pF and Cs = 0.36. As shown in Fig. 5b, the stepped-impedance structures results in a large IL towards the upper edge of the pass-band. Hence, a rectangular structure was added to reduce the IL in the filter’s pass-band. In other words, poles of the open stub resonator should be changed synchronously with SIR. The middle capacitor changes the resonance frequency of the open stub resonator. Here, Cs changes between a range of 0.16 pF to 0.36 pF. Accordingly, Cm values are tuned to achieve low IL and high return loss. Figure 12 shows the frequency response of the proposed filter with multiple values of the Cs and Cm that are optimized and tuned to create a flat pass-band with low IL.

Table 1 shows the values of Cs and its corresponding Cm and band-width or BW. According to the table, when the value of the Cs is low the band-width is high and vice versa. Also Cm and Cs have nonlinear relationship.

**Table 1:** Values of Cs and its corresponding Cm and band-width.

Cs (pF)	Cm	BW (MHz)
0.16	0.5	1600
0.26	0.7	1200
0.36	1.3	600

To quantify the relationship between Cs (pF) and Cm (pF), the curve fitting technique is applied, resulting in a linear relationship as illustrated in (5)

5 $$Cm = 2000*Cs^{4} - 1880*Cs^{3} + 650Cs^{2} - 96*Cs + 2.3$$

There is also a linear relationship between the filter BW (GHz) and Cs (pF) as shown in (6).

6 $${\text{Cs}} = - 0.{2254}*{\text{BW}} + 0.{526}$$

Levenberg–Marquardt optimization algorithm was used to extract coefficients of the polynomials in (5) and (6).

## Results

**Figure 13:**
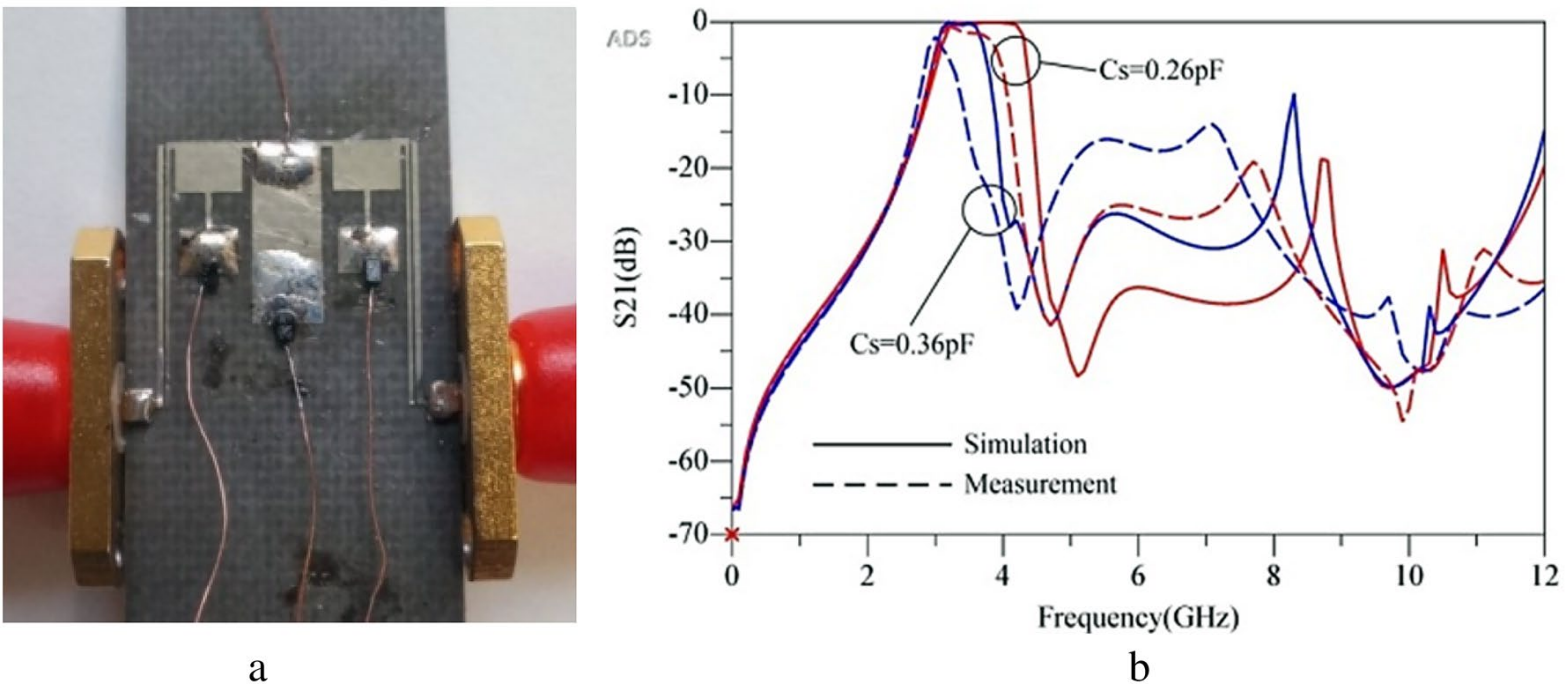
(**a**) Fabricated prototype tunable BPF and (**b**) Simulation results and measurement results.

One prototype of the filter was fabricated and tested on a substrate of Rogers 4003 with a thickness of 31 mil. The substrate is low-cost and proper to lower fabrication cost. The circuit layout was implemented on the substrate top layer using wet photolithography process. In this process, photoresist material coats the top layer and via a mask the layout will be imposed on it. Depending on the type of the photoresist, it will remain soft or get hard when ultraviolet light meets the material. The varactor diodes used in the prototype are SMV2205-040LF. The most important challenge of fabrication process is the minimum feature. A pass-band with low IL needs a coupling with minimum gap. Here fabrication minimum feature is 0.1 mm. The disturbing frequencies are suppressed from 4.2 GHz up to 12 GHz under − 15 dB levels. The BPF illustrates the sharp roll-off rate. The electrical size of the BPF is only 0.258 λ_g_ × 0.255 λ_g_, where λ_g_ is the guided wavelength at the central frequency. Figure 13a and b show the prototype filter and its tunable response, respectively. As measurement result shows, there is a difference between simulation and measurement result. The main reason for such a divergence is that we used only an easy model for varactor diodes, only capacitors as shown in Fig. 10, however parasitic elements for lumped diodes are not ignorable, although discrete circuits has lower quality than monolithic microstrip circuits.

Table 2 draws a comparison between the proposed filter performance and some of the recently published works.

**Table 2:** Comparison between the proposed filter and other works.

Refs	IL (dB)	Size (λ_g_^2^)	Tuning range (GHz)
^8^	3.5	0.013	1.56
^9^	1.5	–	2.7–3.4
^12^	1	–	0.9–2.2
^ 16^	0.5	0.079	1.4–2.9
^18^	2	0.009	2.67–3.78
This work	2	0.066	3.15–6.15

It is shown that the proposed filter has a wide tunable range between 3.15 and 6.15 GHz with compact size of 0.066 λ_g_^2^. To control the band-width of the filter with proper IL and flat pass-band, a relationship between two control parameters which are imposed on the diodes are derived by curve fitting technique. This method is straightforward and practical. In^41^, there is review for reconfigurable filters for 4/5G systems. According to the paper, there are BPFs with IL of 4 dB or even 6 dB of IL According to simulation results and considering ideal PIN diodes as simple capacitors, the proposed filter has an IL of up to 2 dB in 5G bands respectively, which is practical for 5G applications.

## Conclusion

A microstrip varactor tuned BPF with a straightforward design procedure is presented in this work. The main contribution of the paper compared to the others is its IL compensating feature feasible via Compensator diode or Cm that create a better pass-band. Also, two parameters of control are easily applied and their relationships are derived by curve fitting technique. The meandered coupled lines, the open stub and the stepped-impedance structures are easily incorporated in the meandered lines. The proposed structure is suitable for low power applications. The FR band-width or TP_1_ and TP_2_ locations can be easily tuned using the equations extracted from the equivalent circuits and cover a large band-width extending from 3.15 to 6.05 GHz suitable for 3GPP standard channels, including n77, n79 and 5G Wi-Fi. The simulation results verify the filter performance. The filter can suppress all harmonic bands from 4.2 up to 12 GHz. The filter is compact and its size is 0.258 λ_g_ × 0.255 λ_g_, where λ_g_ is the guided wavelength at the central frequency.

## Data Availability

Data generated during the current study will be made available from the corresponding author on reasonable request.
